# Sweet Syndrome and Valley Fever: A Case Report

**DOI:** 10.1016/j.acepjo.2025.100224

**Published:** 2025-07-15

**Authors:** Rachel Meach, Rick McPheeters, Carol Tang, Shikha Mishra

**Affiliations:** 1 Western University of Health Sciences, Pomona, California, USA; 2 Department of Emergency Medicine, Kern Medical, Bakersfield, California, USA; 3 Department of Infectious Disease, Kern Medical, Bakersfield, California, USA

**Keywords:** Erythema sweetobullosum, Sweet’s syndrome, acute febrile neutrophilic dermatosis, Valley fever, Coccidioidomycosis

## Abstract

Sweet’s syndrome, also referred to as erythema sweetobullosum, is a rare dermatologic condition that may serve as a clinical diagnostic clue in the emergency department (ED) for coccidioidomycosis, commonly known as Valley fever. This case describes a patient who presented with the acute onset of painful bullous lesions on the forearms, chest, and anterior legs. Recognition of these cutaneous findings as Sweet’s syndrome, in conjunction with a high index of suspicion for Valley fever, led to the initiation of appropriate treatment with fluconazole and prednisone prior to the return of confirmatory coccidioidomycosis serologies. Early therapeutic intervention resulted in symptomatic improvement.

## 1 Introduction

Coccidioidomycosis, or Valley fever, is a fungal infection associated with significant morbidity and mortality, particularly in endemic regions. Its clinical manifestations range from mild respiratory symptoms to severe, disseminated disease. Early diagnosis is essential to facilitate timely treatment. We present a case in which a patient’s presentation with Sweet’s syndrome—characterized by painful vesiculobullous skin lesions (erythema sweetobullosum)—was instrumental in identifying coccidioidomycosis in the emergency department (ED) setting.^1^

## 2 Case

**Figure 1:**
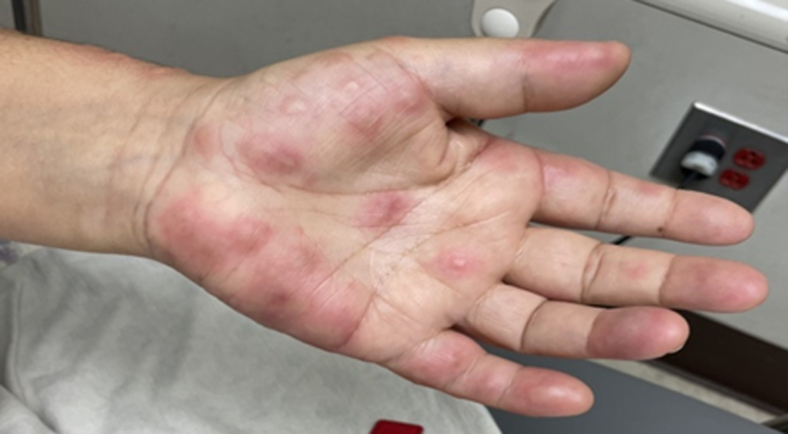
Multiple bullae to left palm in a patient with Valley fever.

**Figure 2:**
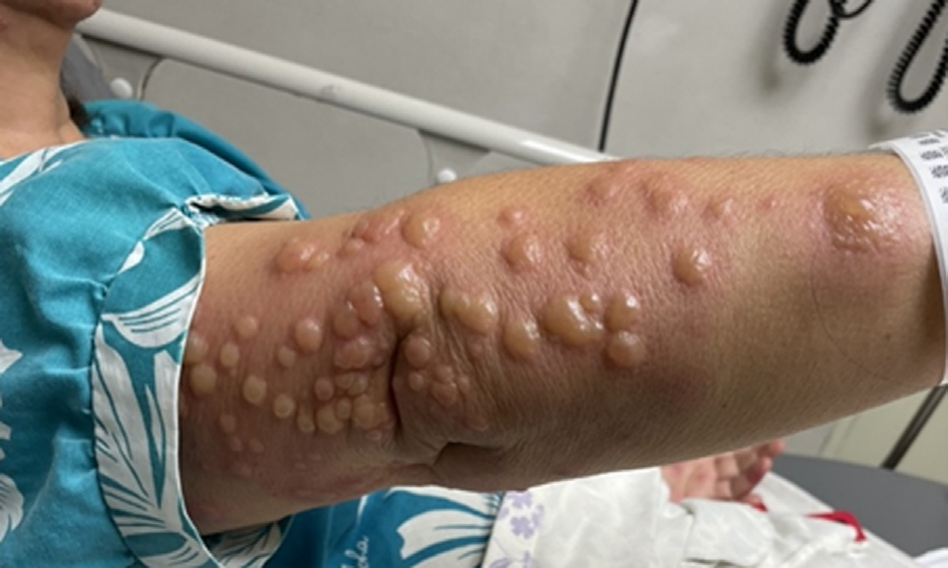
Multiple bullae to the right forearm in a patient with Valley fever.

**Figure 3:**
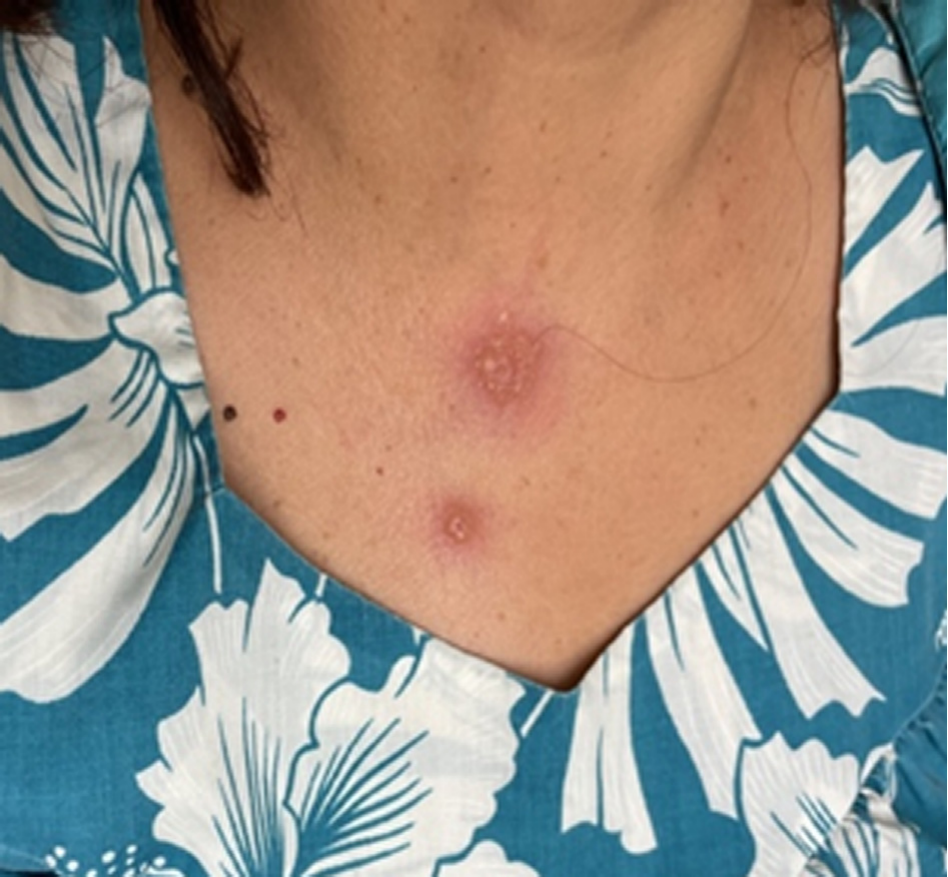
Bullae to the anterior chest wall in a patient with Valley fever.

**Figure 4:**
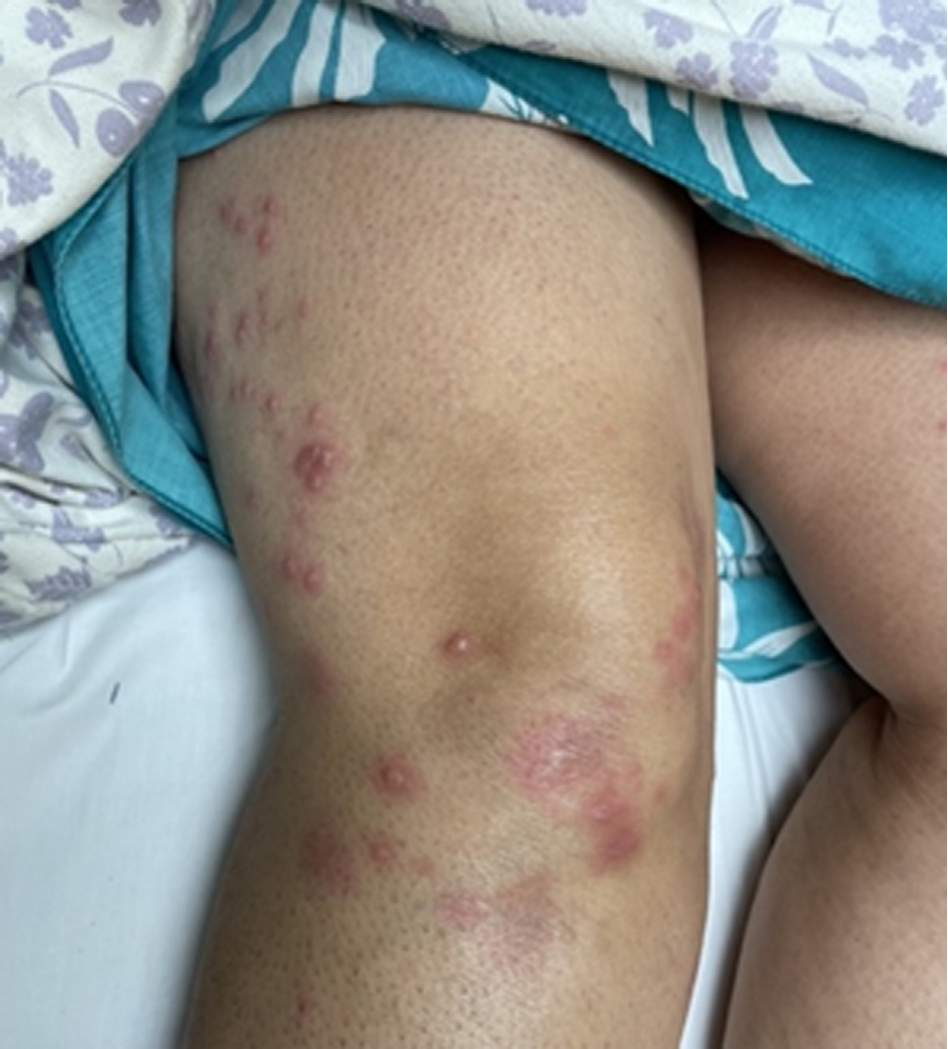
Multiple bullae to the lateral right leg and anterior knee in a patient with Valley fever.

We report the case of a 60-year-old female who presented to the ED with a sudden onset of painful, erythematous bullae affecting the palms (Fig 1), forearms (Fig 2), chest (Fig 3), and lower extremities (Fig 4). The lesions exhibited a negative Nikolsky sign. Her clinical evaluation revealed febrile status and elevated inflammatory markers. A chest radiograph demonstrated a right lower lobe infiltrate. The patient had previously been treated empirically for community-acquired pneumonia without clinical improvement, prompting further evaluation for coccidioidomycosis.^2^^,^^3^

Coccidioidomycosis serologies were obtained in the ED, and empiric antifungal therapy with fluconazole was initiated. Infectious disease follow-up was arranged. Outpatient follow-up confirmed positive serologies for coccidioidomycosis. The patient was continued on fluconazole and prescribed prednisone, resulting in marked clinical improvement. Informed consent for the publication of this case and the accompanying images was obtained from the patient.

## 3 Discussion

Coccidioidomycosis is a fungal infection caused by the inhalation of Coccidioides spores, which are endemic to arid regions of the southwestern United States and parts of Central and South America.^3^ Most infections are subclinical or present with nonspecific flu-like symptoms such as fever, cough, and fatigue. However, more severe presentations, including pneumonia and disseminated disease, may occur. Dissemination can involve the central nervous system, bones, joints, and skin.^4^

The most common cutaneous manifestation of coccidioidomycosis is erythema nodosum—painful, red nodules typically located on the anterior shins. This reaction is thought to represent a delayed hypersensitivity response and can aid in clinical suspicion and early diagnosis.^4^

Sweet’s syndrome, also known as acute febrile neutrophilic dermatosis, is a much rarer cutaneous manifestation. It is characterized by the sudden onset of painful, erythematous plaques or nodules, most often on the upper extremities, face, and trunk. Accompanying systemic features typically include fever and leukocytosis, especially neutrophilia. Sweet’s syndrome has an estimated prevalence of fewer than 1 per 50,000 individuals, and its true incidence is likely underreported due to its rarity. It may be idiopathic or associated with infections, malignancies, or autoimmune conditions. Histologically, it is defined by dense neutrophilic infiltration in the dermis and often responds rapidly to corticosteroids.^5^

In distinguishing Sweet’s syndrome from erythema nodosum, both conditions present with tender cutaneous lesions and may be associated with systemic symptoms, such as fever and leukocytosis. However, erythema nodosum typically presents as deep, erythematous, subcutaneous nodules most often localized to the anterior shins, and it is considered panniculitis.^4^ In contrast, Sweet’s syndrome presents with more superficial, edematous, and often bullous plaques or nodules. In our case, the rapid onset of painful, vesiculobullous lesions on the extremities and trunk with a negative Nikolsky sign and absence of subcutaneous nodularity favored a diagnosis of Sweet’s syndrome. Although skin biopsy and histopathologic examination are considered the gold standard for diagnosing Sweet’s syndrome, a biopsy was not pursued in this case due to the classic clinical presentation and the patient’s rapid improvement with corticosteroids. Although histopathology is helpful in ambiguous presentations, its utility may be limited when clinical features are definitive and response to therapy is prompt.^5^^,^^6^

Recent literature continues to highlight the rare but clinically relevant association between Sweet’s syndrome and acute pulmonary coccidioidomycosis. A 2024 case series by Batchinsky et al^7^ described patients in West Texas presenting with Sweet’s syndrome in the setting of acute pulmonary coccidioidomycosis, further supporting the role of this distinctive dermatologic manifestation as an early clinical clue in endemic areas. Their findings underscore the importance of recognizing erythema sweetobullosum as a potential immune-mediated marker of underlying fungal infection and initiating appropriate diagnostic evaluation and treatment even prior to confirmatory serologies.^8^

Although Sweet’s syndrome is classically idiopathic, it is essential to consider a broad differential diagnosis when patients present with painful erythematous plaques or nodules, particularly in the ED. Sweet syndrome may occur as a reactive phenomenon secondary to infections, autoimmune conditions, malignancies—especially hematologic cancers—and certain medications such as granulocyte colony-stimulating factor, antibiotics (eg, trimethoprim-sulfamethoxazole), or antiepileptics. Korkut et al^9^ presented a case emphasizing Sweet’s syndrome as a dermatologic emergency secondary to a bacterial upper respiratory tract infection, highlighting the importance of evaluating recent infection history when considering this diagnosis. Similarly, Pulido-Pérez and Bergon-Sendin^10^ described a patient with Sweet’s syndrome associated with an underlying hematologic malignancy, illustrating the need to investigate neoplastic causes in the appropriate clinical context.

In contrast, our case exemplifies Sweet’s syndrome as a reactive cutaneous manifestation of a fungal infection—coccidioidomycosis. The patient had no evidence of drug exposure, malignancy, or autoimmune disease that would otherwise explain the dermatologic findings. Instead, her presentation and rapid improvement with corticosteroids and antifungal therapy, combined with subsequent positive coccidioidomycosis serologies, strongly support an infectious etiology. Recognizing the wide range of potential causes is essential for clinicians to avoid premature closure and to guide targeted workup and management.

## Funding and Support

By *JACEP Open* policy, all authors are required to disclose any and all commercial, financial, and other relationships in any way related to the subject of this article as per ICMJE conflict of interest guidelines (see www.icmje.org). The authors have stated that no such relationships exist.

## Conflict of Interest

All authors have affirmed they have no conflicts of interest to declare.
